# The Deconstructed Granuloma: A Complex High-Throughput Drug Screening Platform for the Discovery of Host-Directed Therapeutics Against Tuberculosis

**DOI:** 10.3389/fcimb.2018.00275

**Published:** 2018-08-14

**Authors:** Lu Huang, Nicole L. Kushner, Monique E. Theriault, Davide Pisu, Shumin Tan, Case W. McNamara, H. Mike Petrassi, David G. Russell, Amanda C. Brown

**Affiliations:** ^1^Department of Microbiology and Immunology, College of Veterinary Medicine, Cornell University, Ithaca, NY, United States; ^2^Department of Molecular Biology and Microbiology, Tufts University School of Medicine, Boston, MA, United States; ^3^California Institute for Biomedical Research (Calibr), La Jolla, CA, United States

**Keywords:** tuberculosis, pulmonary, *Mycobacterium tuberculosis*, high-throughput screening assays, host-directed therapeutics, macrophages

## Abstract

*Mycobacterium tuberculosis* (Mtb) continues to be a threat to Global Public Health, and its control will require an array of therapeutic strategies. It has been appreciated that high-throughput screens using cell-based assays to identify compounds targeting Mtb within macrophages represent a valuable tool for drug discovery. However, the host immune environment, in the form of lymphocytes and cytokines, is completely absent in a chemical screening platform based on infected macrophages alone. The absence of these players unnecessarily limits the breadth of novel host target pathways to be interrogated. In this study, we detail a new drug screening platform based on dissociated murine TB granulomas, named the Deconstructed Granuloma (DGr), that utilizes fluorescent Mtb reporter strains screened in the host immune environment of the infection site. The platform has been used to screen a collection of known drug candidates. Data from a representative 384-well plate containing known anti-bacterial compounds are shown, illustrating the robustness of the screening platform. The novel deconstructed granuloma platform represents an accessible, sensitive and robust high-throughput screen suitable for the inclusive interrogation of immune targets for Host-Directed Therapeutics.

## Introduction

*Mycobacterium tuberculosis*, the causative agent of human tuberculosis (TB), poses an ongoing threat to human health. The escalating incidence of drug resistant strains provides a new impetus to understand this complex pathogen and to identify novel therapeutics. Ginsberg and Spigelman (Ginsberg and Spigelman, 2007) list the goals and challenges for improved TB therapy, one of which is to “discover and develop new drugs that have novel mechanisms of action and are effective against persistent bacilli,” as well as to “develop new preclinical approaches to identify optimized drug combinations….” As a first step toward these aims, we have directed our efforts to developing improved drug screening tools. Traditional drug discovery programs for the identification of new anti-microbial compounds have focused on either target-based screening against enzymatic activities that have been shown genetically to be essential for bacterial survival (Mdluli and Spigelman, 2006; Lamichhane, 2011), or phenotypic screens exploring bacterial survival in rich broth (Collins and Franzblau, 1997; Pethe et al., 2010). Both approaches have had limited success in recent times, calling into question whether we should continue to devote our major efforts down these paths. Whilst complicated, the most frequently cited reasons for the failure of these approaches are limited drug permeability in the former approach, and the inappropriate nature of rich bacterial broth to mirror the physiology within the host in the latter approach (Koul et al., 2011; VanderVen et al., 2015; Zuniga et al., 2015).

There is also the sense that the “low-hanging fruit” have already been picked, and compounds with known modes of action continue to re-emerge from ongoing screens. For these reasons we feel that it is critical to develop new, innovative approaches to drug discovery that explore and exploit the host-derived pressures on *Mycobacterium tuberculosis* (Mtb) to identify compounds with enhanced efficacy within the host.

Host-direct therapy (HDT) is an emerging theme in the treatment of infectious diseases; small molecules are utilized to modulate or perturb host responses, to reduce replication or persistence of pathogens, to limit tissue damage, and to enhance the efficacy of current treatments (Kim and Yang, 2017; Kolloli and Subbian, 2017). Current HDT approaches for Mtb target an array of pathways, including: disintegration of granuloma structure, autophagy, anti-inflammatory response, and, more recently, checkpoint blockage to unleash the immune suppression induced by Mtb infection in the lung (Kolloli and Subbian, 2017; Kaufmann et al., 2018). However, our knowledge of immune protection against TB is heavily reliant on studies based on failed immunity in genetically-modified animals and catastrophic human genetic lesions, and this has likely limited the development of both vaccines and new host-dependent therapies (Huang and Russell, 2017). Recent work has indicated that, in addition to immune-mediated control, host macrophage ontogeny also plays a major role in expansion of bacterial numbers and disease progression (Huang et al., 2018). Furthermore, the efficacy of existing anti-tuberculosis drugs *in vivo* is markedly different from their *in vitro* activity, and is impacted by the immune status of the host tissue and cellular environment (Liu et al., 2016; Russell, 2017). These layers of host-dependent complexity represent an increased challenge to development of effective new drugs, but they also represent an un-mined opportunity if we can develop appropriate *in vitro* screens that incorporate this biology. Therefore, to accelerate anti-TB drugs and HDT discovery, we need a discovery platform with minimal assumptions that provides a more holistic representation of the complex biology of the infected host.

With this aim in mind we have developed a high throughput screening (HTS) assay utilizing cells recovered from Mtb-infected mouse lungs (*D*econstructed *Gr*anuloma, DGr), which we are using to screen compounds with efficacy against Mtb within the context of host-derived immune cells. These cells, which comprise mainly of resident alveolar macrophages, monocyte-derived macrophages, neutrophils and T- and B-lymphocytes, have been recruited and educated by the infection environment and therefore reflect the host immune pressures active within the TB granuloma. The DGr platform has been adapted to an HTS-compatible, 384-well format and generates a robust reproducible readout with an extensive dynamic range. We believe that this novel HTS platform will reveal new avenues toward the manipulation of the host immune environment to restrict bacterial growth and survival, and to act in concert with existing drug treatments and increase their *in vivo* potency.

## Materials and methods

### Mice

C57BL/6 mice were purchased from The Jackson Laboratory. Mice were used at 6–8 week old. All mice were maintained in a specific pathogen-free biosafety level-3 facility at Cornell University. Animal care was in accordance with the guidelines of the Association for Assessment and Accreditation of Laboratory Animal Care. All animal procedures were approved by the Institutional Animal Care and Use Committee of Cornell University.

### Mtb cultivation

Mtb strains were cultured in Middlebrook 7H9 liquid medium supplemented with 10% v/v OADC (oleic acid, bovine serum albumin, D-glucose, catalase, sodium chloride; Becton Dickinson) and 0.05% w/v Tyloxapol, or on solid Middlebrook 7H10 agar supplemented with 10% v/v OADC at 37°C, with 25 μg/ml kanamycin or 30 μg/ml hygromycin B where appropriate. Cultures for HTS were grown for 5 days in 100 ml roller cultures (7H9/OADC/Tyloxapol).

### Mtb reporter strains

The Erdman background of Mtb was used to generate all reporter strains, this is a reference strain commonly used for studies of pathogenesis. We chose to use two different fluorescent reporter strains for the initial infection (mCherry) and the *ex-vivo* challenge (mKO) to allow us to identify cells infected from each of the separate infections. We found that after incubation, following the *ex-vivo* challenge, the intensity of mKO signal increased, however the mCherry readings remained constant. We favor mKO as a reporter for the final plate assay over mCherry because it has a greater range/sensitivity and a lower background. (1) mCherry strain: For mouse infection we used a *smyc'*::mCherry strain, which has been previously described (Tan et al., 2013); mice were inoculated intranasally with ~1,000 CFU of Erdman(*smyc'*::mCherry) in 30 μl PBS containing 0.05% Tween 80. The inoculum dosage was confirmed by plating different dilutions on 7H10 plates. Plates were incubated at 37°C and colonies enumerated after 3 weeks. (2) monomeric Kusabira Orange (mKO) strain: For the pilot HTS screen we created a tetracycline (tet) inducible mKO Mtb reporter strain, Erdman(P_606_'::mKO-tetON), by introduction of a replicating plasmid containing the tet-ON regulator upstream of P_606_ driven mKO, in the destination Gateway vector pDE43-MEK. The fluorescent protein Kusabira Orange was originally isolated from the stony coral *Fungia concinna* (Karasawa et al., 2004), and the mKO derivative used here was codon-optimized for Mtb by GenScript. (3) Dual mCherry-mKO strain: For imaging purposes we generated a dual reporter strain, Erdman(P_606_'::mKO-tetON, *smyc*'::mCherry), which constitutively expressed the mCherry fluorescent protein under the *smyc* promoter (Tan et al., 2013), and also inducibly expressed mKO upon the addition of exogenous tetracycline, on the replicating Gateway plasmid pDE43-MEK backbone.

### BMDM and J774 preparation

BMDM were isolated from C57BL/6J mice and cultured in DMEM (Corning Cellgro) supplemented with 10% FBS (Thermo Scientific), 20% L929-conditioned media, 2 mM L-glutamine, 1 mM sodium pyruvate, and 1% penicillin/streptomycin (Corning Cellgro) at 37°C, 5% CO_2_, for 9 days prior to seeding for experiments. J774 cells were obtained from ATCC. Cells were maintained in DMEM +10% FBS + 2 mM L-glutamine + 1 mM sodium pyruvate (complete DMEM) + 1% penicillin/streptomycin at 37°C, 5% CO_2_, until 90% confluence, in T300 flasks. Cells were scraped from the flasks into cold, sterile PBS, centrifuged at 700 rpm for 10 mins, and re-suspended at 1 x 10^6^/ml in complete DMEM. 30 μl of the cell suspension was then seeded into each well of 384-well optically clear, black plates (~3 × 10^4^ cells/well) and incubated overnight at 37°C, 5% CO_2_.

### Generation of deconstructed granuloma (DGr) cell suspensions

Mice were infected with Mtb, and sacrificed after 3 weeks. In the actual drug screen we process and pool cells from 5 infected mice to generate 6 × 384 well plates. The pooling of the granuloma cells minimizes variation and renders the screen much more reproducible from run to run by limiting mouse-to-mouse variation. Mouse lungs lungs were removed and placed into Miltenyi GentleMACS C-tubes, containing 5 ml lung dissociation buffer (5% FBS/PBS solution containing 250 U/ml collagenase IV and 20 U/ml DNase). Lungs were processed using the GentleMACS Lung 37C_m_LDK_1 program, with 1 set of lungs per tube. Cells were then pooled and re-suspended in 10 ml 5% FBS/PBS per set of lungs, passed through a 70 μm cell strainer, and then pelleted at 1,200 rpm for 10 min. 3 ml of ACK buffer (Lonza) was used to re-suspend the cell pellet and incubated at room temperature for 5 min to lyse red blood cells, following which 10 ml 5% FBS/PBS was added and the cell suspension passed through a 40 μm cell strainer. After a final spin at 1,200 rpm for 10 min the resulting pellet was re-suspended in 20 ml complete DMEM and cell counts performed. The cells were diluted to 3.33 × 10^6^/ml in complete DMEM with Amphotericin B (final concentration 20 μg/ml). 30 μl of cells were seeded into each well of a 384 well optically clear, black plates (~10^5^ cells/well). Lung cells were then followed by a secondary *ex vivo* infection with a desired fluorescent Mtb reporter strain at MOI 0.1 as detailed below.

### Test compounds

Test drugs were arrayed from a 2 mM stock solution in DMSO via acoustic dispense (ECHO 550 liquid handler; Labcyte Inc.) into 384-well Axygen P-384-120SQ-C-S plates, sealed and stored frozen prior to use. Each well contained a final volume of 500 nl 1.5 mM compound. Pre-spotted assay plates were thawed and 24.5 μl of incomplete DMEM was added to each well to give a final drug concentration of 30 μM/well. These compound plates were centrifuged at 1,000 rpm and stored at 4°C prior to addition to assay plates.

### High-throughput screen protocol

Erdman(P_606_'::mKO-tetON) was grown for a week in 100 ml supplemented Middlebrook 7H9 broth with kanamycin. On the day of infection, 50 ml of culture was harvested by centrifugation at 3,000 rpm for 10 mins, re-suspended in 1 ml sterile cold basal uptake buffer (25 mM dextrose, 0.5% bovine serum albumin, 0.1% gelatin (Sigma), 1 mM CaCl_2_, 0.5 mM MgCl_2_ in PBS), passed through a tuberculin needle 6 times, and re-suspended in complete DMEM media at a final OD_600_ of 0.2. DGr cells were then infected with 10 μl/well of this bacterial suspension using a Perkin Elmer Janus liquid handling robot. Plates were returned to incubator (37°C, 5% CO_2_) for 1 hr to allow bacteria uptake by host cells. Following this incubation, the DGr cells received 10 μl /well complete DMEM with 60 μg/ml Amphotericin B (final concentration in assay 20 μg/ml) and 0.075 μg/ml isoniazid (INH), where appropriate (final concentration in assay 0.0125 μg/ml). Plates were returned to the incubator following this addition. Finally, test inhibitor compounds were transferred to the assay plates, with each well receiving 10 μl of 30 μM test compound (final compound concentration 5 μM). Plates were incubated at 37°C, 5% CO_2_. On day 4, 10 μl of 1.4 μg/ml anhydrotetracycline (ATc) was added per well (final concentration 200 ng/ml). Assay plates were returned to the incubator at 37°C, 5% CO_2_ for a further 3 days. On day 7, the fluorescence was read from the bottom on a Perkin Elmer Envision plate reader—excitation 530/8 nm, dichroic 555 nm, emission 579/25 nm.

The inclusion of Amphotericin B is a potential confounder because of its known immune-modulatory capacity (Hedges et al., 2015) but, because the lung is not a sterile environment, we needed to add the Amphotericin B as an antifungal. We have a series of counter screen assays to verify and validate hits from the primary screen therefore spurious positives will be removed from those compounds advanced in further analysis.

### Flow cytometry

Lung cells were incubated with Fc block (eBioscience) for 15 min. Cells were then counted and incubated for 30 min in the dark with fluorophore-conjugated antibodies. Fluorophore-conjugated mAb specific to mouse CD45 (30-F11; BD Biosciences), CD11b (M1/70; BD Biosciences), CD64 (X54-5/7.1; Biolegend), CX_3_CR1 (SA011F11; BioLegend), Ly6G (1A8; BioLegend), MerTK (DS5MMER), SiglecF (E50-2440; BD Biosciences), CD4 (GK1.5), EpCAM (G8.8) were purchased from eBioscience unless otherwise indicated. Fixable viability dye was purchased from eBioscience. Cells were analyzed with a Becton Dickenson LSRII flow cytometer and data analyzed with FlowJo software (Tree Star).

### Confocal microscopy

Cells were imaged using a Leica SP5 confocal microscope and images were exported and analyzed using Volocity software (PerkinElmer).

### Statistical analysis

Results were statistically analyzed using Student's *t*-test, or one-way ANOVA test with multiple comparisons where appropriate, with Prism 6.0 (GraphPad Software).

## Results and discussion

### Increased number of infected myeloid cells after challenge with fluorescent Mtb

**Figure 1:**
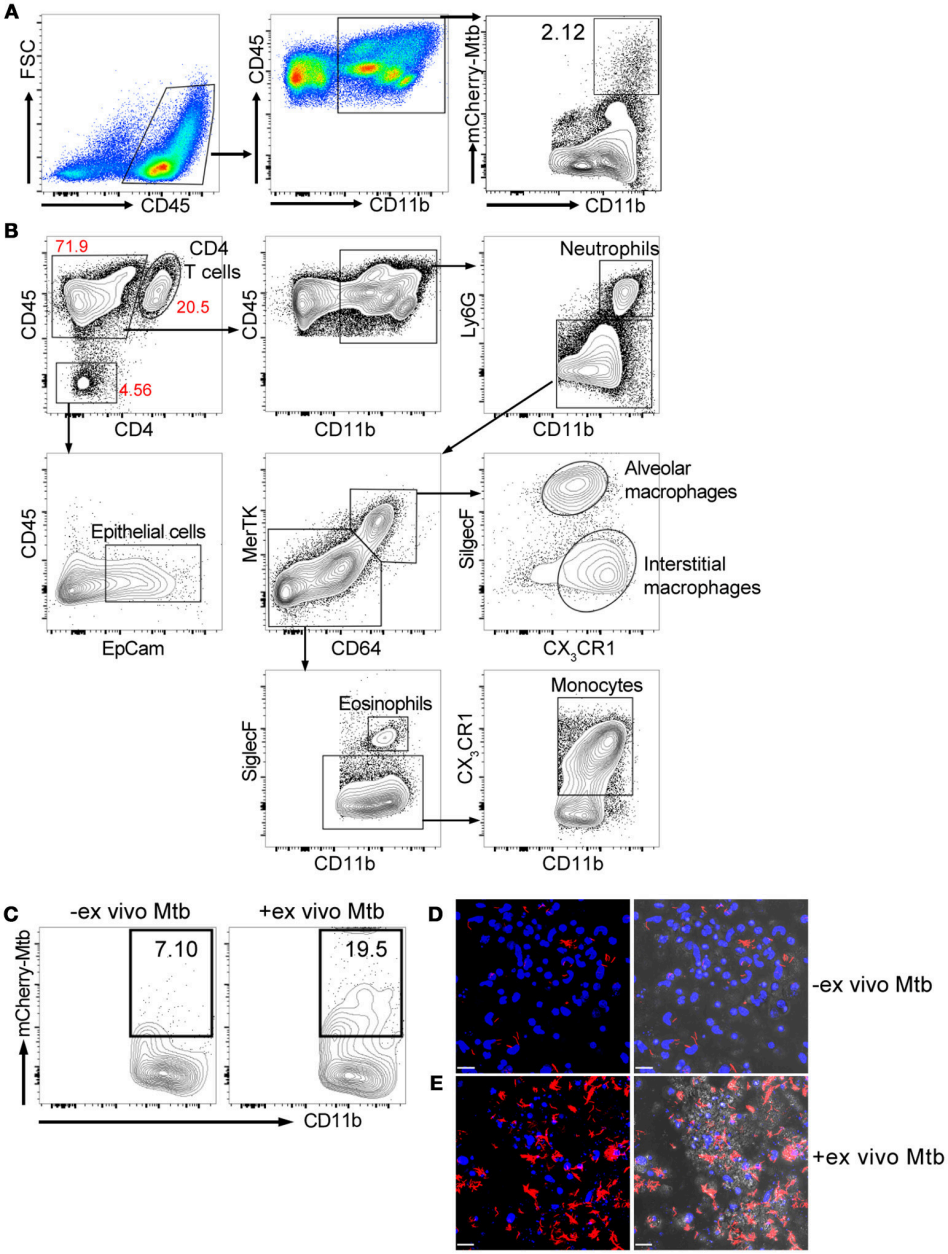
*Ex vivo* challenge with the fluorescent Mtb strain increases the numbers of infected myeloid cells. Mice were infected with ~1,000 CFU Erdman(*smyc'*::mCherry) for 3 weeks. Lung single cell suspensions were isolated for the analysis. **(A)** Gating strategy and flow cytometric analysis of percentage of Mtb-infected myeloid cells (CD45^+^CD11b^+^) in the mouse lung after 3 weeks infection. **(B)** Multi-color flow cytometry analysis of myeloid populations in Mtb infected mouse lung at 3 weeks post infection. **(C)** Flow cytometry analysis of percentage of Mtb-infected lung myeloid cells before and after *ex vivo* challenge with mCherry Mtb at MOI 0.1 for 24 h. **(D,E)** Confocal images of cultured lung cells isolated from mCherry Mtb-infected mice without **(D)** or with **(E)** *ex vivo* challenge. The left panels of **(D,E)** show the fluorescent images and the right panels show the same fluorescent images overlaid with the brightfield images. Scale bar = 25 μm in **(D,E)**. The experiments were repeated at least two times.

We isolated lung cells from C57BL/6J mice challenged with 1000 CFU of Erdman(*smyc'*::mCherry) for 3 weeks, to examine the distribution of mCherry Mtb in the infected mouse lung, and investigate whether the fluorescent bacterial signal in the isolated lung cells could be detected by a plate reader. Three weeks post infection was chosen because this is the transition period from innate immunity to the initiation of adaptive immunity in the lung during Mtb infection (Urdahl, 2014). We found that although Mtb predominately infects CD45^+^CD11b^+^ myeloid cells, only 2.12% of the CD45^+^CD11b^+^ cells carried Mtb (Figure 1A). We also developed a comprehensive multi-color flow cytometry analysis and further revealed different myeloid populations in the Mtb infected murine lung, including neutrophils, monocytes, alveolar and interstitial macrophages (Figure 1B). The tissue homogenization process releases these immune cells preferentially, and we observe only a relatively modest contribution from epithelial lung cells, fewer than 5% CD45- cells, in the resulting suspension (Figure 1B). Unfortunately, mCherry fluorescence was below the level of detection by the plate reader due to the low penetrance of bacteria in the lung myeloid cell population. To overcome this issue, we subjected the cells to an *ex vivo* challenge with reporter Mtb to further enhance the signal. Indeed, the percentage of mCherry Mtb infected CD45^+^CD11b^+^ cells was significantly increased 3 days post *ex vivo* challenge (Figure 1C), and this was further confirmed by confocal microscopy (Figures 1D,E). We conclude that the lung myeloid cells isolated from Mtb-infected mice are highly phagocytic, enabling an *ex vivo* challenge to markedly increase the number of infected cells and enhance fluorescent signal.

### Validation of tet-ON inducible Mtb reporter strain

**Figure 2:**
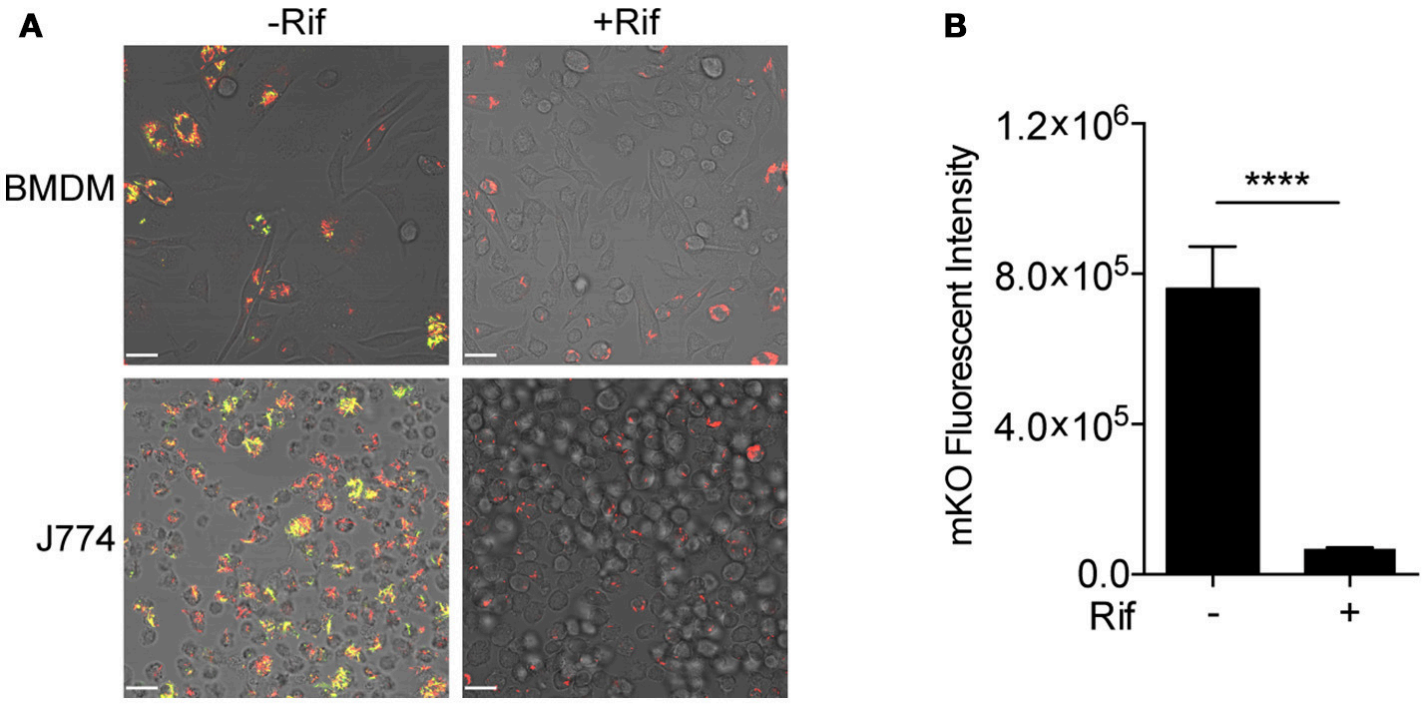
Validation of tet-ON inducible Mtb strain. **(A)** Confocal images of tet-ON inducible mKO/mCherry infected BMDM (MOI 3) and J774 (MOI 4) cells with or without Rif treatment. Rif (5 μM) was added to Mtb infected cells 3 h post infection. ATc (200 ng/ml) was added to cultures on day 4, and confocal images were taken on day 7. **(B)** The fluorescence signal of mKO was obtained with a plate reader. Data are shown as mean ± standard deviation. The experiments were repeated at least two times. *P*-value was calculated using Student's *t*-test. ****P < 0.0001.

Fluorescent reporter-based assays have been used extensively to test and screen antimicrobial compounds against mycobacteria (Stanley et al., 2012; VanderVen et al., 2015; Gupta et al., 2017). To achieve a higher sensitivity and an extended dynamic range, we constructed a dual inducible/constitutive fluorescent Mtb strain that constitutively expresses mCherry, and expresses mKO upon induction by tetracycline (or its derivative ATc), to enable the specific detection of viable bacteria. The rationale behind the construction of this strain is consistent with previous reports on the use of inducible fitness reporters in mycobacterium (Martin et al., 2012; Mouton et al., 2016). The performance of the new Mtb reporter strain was tested in murine bone marrow-derived macrophages (BMDM) and in J774 cells, a murine macrophage-like cell line. Both mCherry and mKO signal were readily detected by confocal microscopy upon ATc induction of infected cells. In contrast, mKO signal was completely absent in the presence of the anti-tuberculosis drug rifampicin (Rif) in both BMDM and J774 cells (Figure 2A). Analysis of the mKO fluorescence signal intensity with a plate reader demonstrates the robust dynamic range of the reporter strain in J774 cells, in the presence or absence of Rif (Figure 2B). This new inducible Mtb reporter strain therefore represents a highly sensitive tool suitable for HTS.

### Deconstructed granuloma model in 96- and 384-well platforms

**Figure 3:**
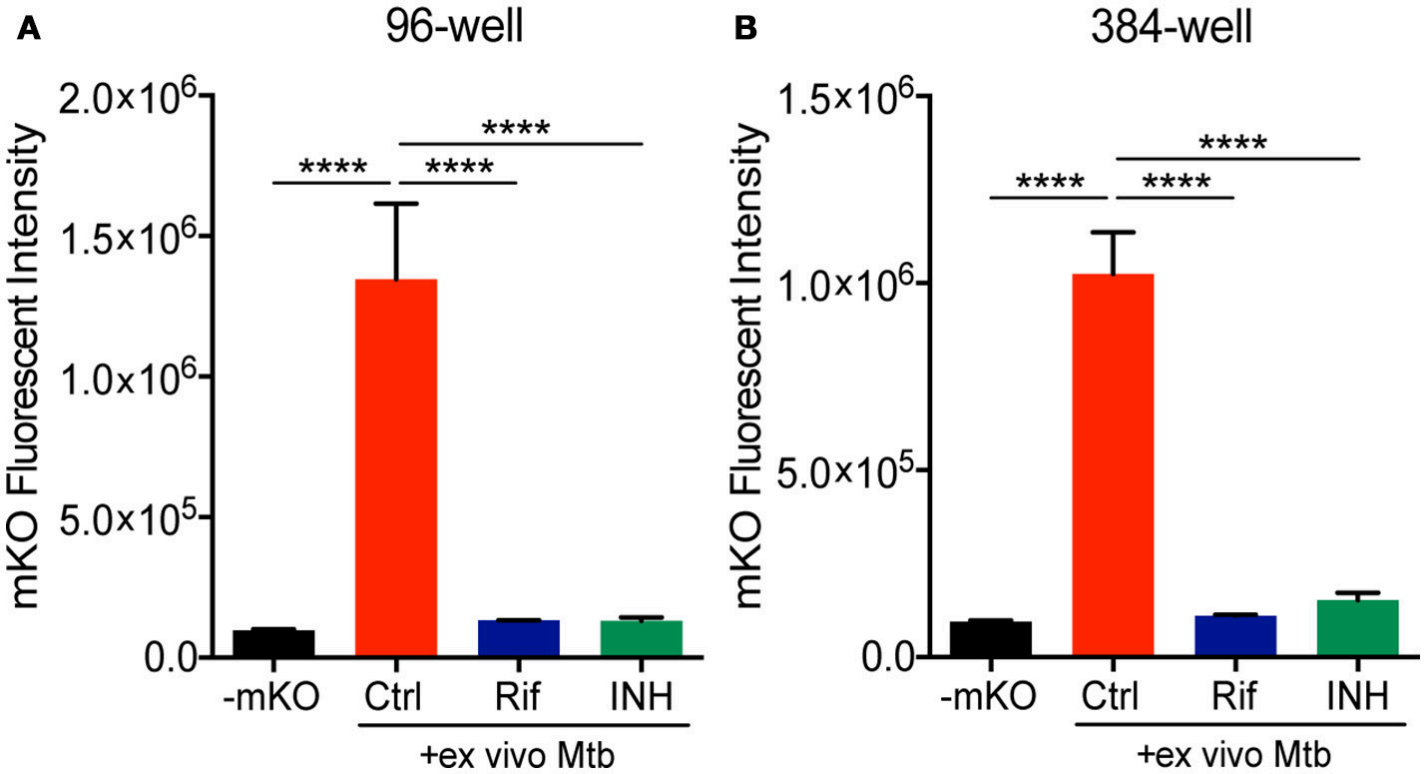
Deconstructed granuloma assay in 96- and 384-well platforms. Lung cells isolated from infected mice were cultured in **(A)** 96-well or **(B)** 384-well plates. Tet-ON inducible mKO/mCherry Mtb were added to the cultures at MOI 0.1 with or without addition of Rif and INH. The fluorescence signal of mKO was acquired with a plate reader. Data are shown as mean ± standard deviation. The experiments were repeated at least two times. *P*-value was calculated using one-way ANOVA test with Tukey's multiple comparisons. ****P < 0.0001.

We next combined the *ex vivo* challenge with a inducible tet-ON mKO reporter to validate the assay in 96- and 384-well plates and test its performance in a HTS format. We created single cell suspensions of the lungs removed from mice infected for 3 weeks with mCherry Mtb. These cells include the large numbers of innate immune cells that infiltrate into the lung, along with the developing lymphocyte response. The mKO Mtb strain was added *ex vivo* to lung cell cultures established in 96- or 384-well plates. Both Rif and INH were included in the test plates in control wells. Upon ATc induction, mKO signal was detected by the plate reader in the control wells but not in the control wells containing anti-tuberculosis drugs (Figure 3). The magnitude of the response likely reflects both bacteria numbers and bacterial fitness (ability to detect and respond to ATc).

### Analysis and presentation of data from the primary drug screen

**Figure 4:**
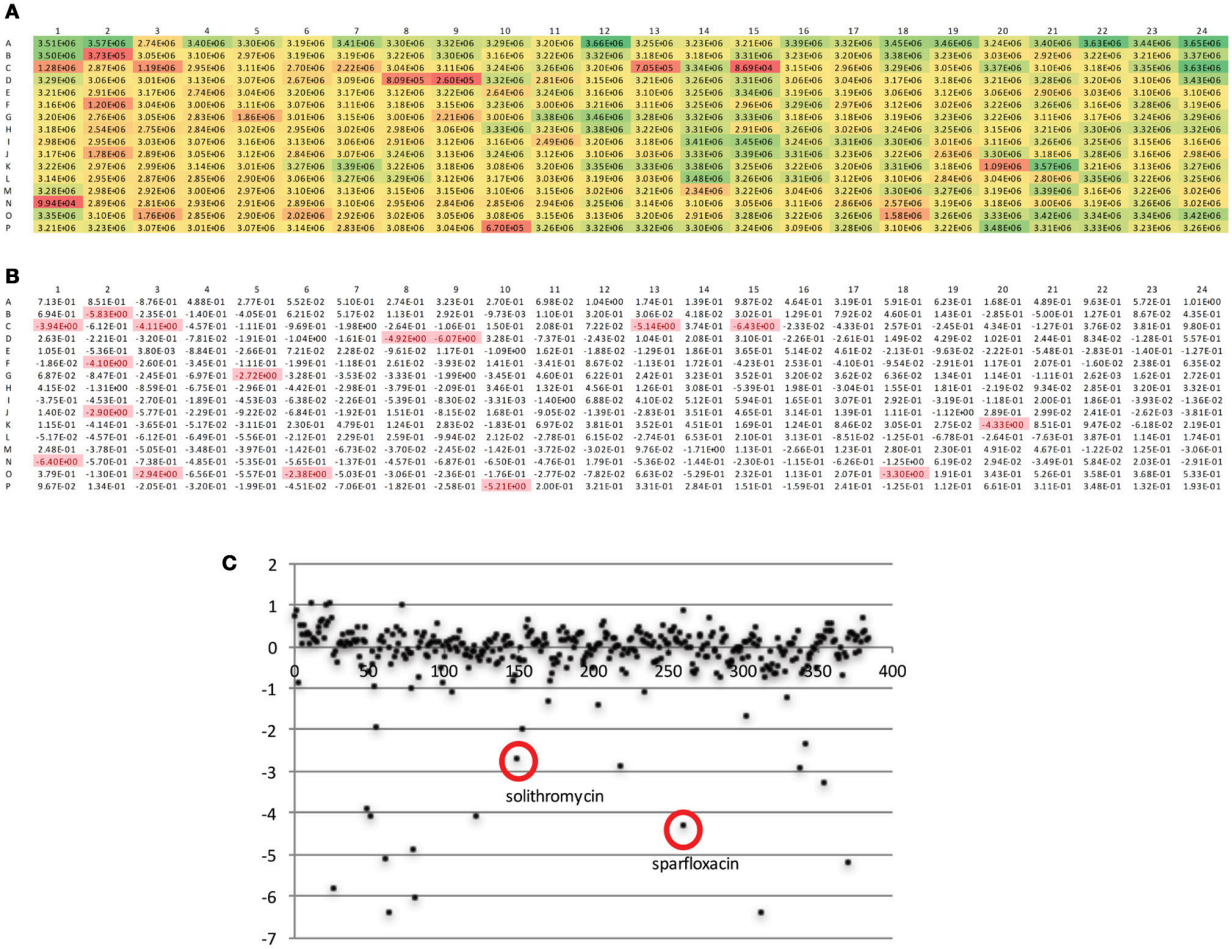
Analysis of fluorescent readout data from a representative 384-well plate. **(A)** Raw fluorescent data as obtained from plate read. **(B)** Data from **(A)** transformed into z-scores, calculated as *z* = x – m/s where m = median of sample plate, and s = sample standard deviation; a z-score of −2 or lower was considered to indicate a “hit.” **(C)** Scatter plot of z scores, x-axis is compound number (1-384), known anti-bacterials within this compound plate are circled.

The DGr has been utilized in a single dose drug screen of 10,000 bioactive compounds, where each compound was tested at 5 μM against the tet-ON mkO strain. The raw fluorescent data from a representative 384-well plate is shown in Figure 4A. These data were transformed into z-scores, calculated as z = x – m/s where m = median of sample plate, and s = sample standard deviation; a z-score of −2 or lower was considered to indicate a “hit” (Figure 4B). Known anti-bacterial compounds present in this particular plate e.g., solithromycin and sparfloxacin, (Figure 4C) were included to validate the platform. Both of these compound classes have been reported to have activity, albeit less than frontline drugs, against Mtb (Rastogi et al., 2000; Pranger et al., 2011). These data demonstrated that the DGr platform represents a viable, sensitive, and robust method for screening compounds for anti-tuberculosis activities. The inclusion of all the immune cell subsets from the infection site in the assay suggests that this platform has the capacity to identify anti-tuberculosis activities that are dependent on a diverse array of host pathways.

Hits from the screen that we have completed have to be validated, and modes of action have to be determined, nonetheless we see considerable value in documenting the DGr platform in depth because this approach has broad application for the interrogation of chemical, genetic and immunological perturbation of the host components from the Mtb site of infection.

## Concluding remarks

We have established an *in vitro* drug screening platform that incorporates the diverse cell populations that are recruited and educated within the murine TB granuloma. In contrast to existing screens on Mtb-infected macrophage mono-cultures, which have already proven useful (Christophe et al., 2010; Stanley et al., 2012; VanderVen et al., 2015), this new platform encompasses the broad range of immune pressures active in *in vivo* infection and therefore provides an increased number of potential targets for host-directed therapeutics. Although previous assays on heterologous cell types challenged *in vitro* have been detailed (Silva-Miranda et al., 2015), this current platform utilizes cells selected and programed by *in vivo* infection, thus making fewer assumptions regarding cell type and immune status. We have demonstrated that the platform is amenable to a HTS in a 384 well plate format and have called it the DGr (*D*econstructed *Gr*anuloma) platform. Through the development process we found that an *ex vivo* challenge was required to amplify the bacterial signal for detection by plate reader. During optimization of the platform we used two different fluorescent reporter Mtb strains: a mCherry strain was used for the initial mouse infection, to generate the immune response in the animal, followed by *ex vivo* infection with a tet-ON mKO strain, employed for detection purposes in the HTS assay. We have performed a HTS screen with this DGr platform and provide data from a representative plate to demonstrate the robustness of the assay. Final assessment of the platform and its value as an HTS to discover novel HDTs is dependent on the analysis of the “hits” from the screening of this compound collection, which is ongoing.

In summary, we have developed a robust new, biologically-rich platform for screening for compounds with anti-tuberculosis activities. We believe that this complex platform encompasses much of the immune environment of the infection site, and is of particular value in the pursuit of Host-Directed Therapeutics. We also suggest that similar approaches using cells dissociated from the infected tissue harvested from experimental *in vivo* infections with other microbial pathogens might have comparable value to other drug discovery efforts.

## Author contributions

LH and DR conceived the study, conducted the initial experiments and interpreted data; LH, NK, MT, and AB were responsible for conducting the experiments, data acquisition and interpretation. ST conceived of the use of mKO in Mtb, and generated and carried out initial tests on the inducible mKO strain. DP provided critical reagents. CM and HP provided the compound collection and assisted in the design and interpretation of the screen data. LH, AB and DR wrote the manuscript. All authors critically read and edited the manuscript.

### Conflict of interest statement

The authors declare that the research was conducted in the absence of any commercial or financial relationships that could be construed as a potential conflict of interest.
